# *Klebsiella pneumoniae* Bacteremia and Capsular Serotypes, Taiwan

**DOI:** 10.3201/eid1706.100811

**Published:** 2011-06

**Authors:** Chun-Hsing Liao, Yu-Tsung Huang, Chih-Cheng Lai, Cheng-Yu Chang, Fang-Yeh Chu, Meng-Shiuan Hsu, Hsin-Sui Hsu, Po-Ren Hsueh

**Affiliations:** Author affiliations: Far Eastern Memorial Hospital, Taipei, Taiwan (C.-H. Liao, C.-C. Lai, C.-Y. Chang, F.-Y. Chu, M.-S. Hsu, H.-S. Hsu);; National Taiwan University College of Medicine, Taipei (Y.-T. Huang, P.-R. Hsueh)

**Keywords:** Klebsiella pneumoniae, bacteremia, bacteria, capsular serotypes, outcome, Taiwan, dispatch

## Abstract

Capsular serotypes of 225 *Klebsiella pneumoniae* isolates in Taiwan were identified by using PCR. Patients infected with K1 serotypes (41 isolates) had increased community-onset bacteremia, more nonfatal diseases and liver abscesses, lower Pittsburgh bacteremia scores and mortality rates, and fewer urinary tract infections than patients infected with non–K1/K2 serotypes (147 isolates).

*Klebsiella pneumoniae* bacteria cause a variety of infections (1,2). Geographic differences in this organism have been recognized, and a high prevalence of liver abscesses has been observed for >20 years in persons in Taiwan infected with *K*. *pneumoniae* (3,4). K1 and K2 are the major capsular serotypes that cause liver abscesses and have increased virulence (4–7). In contrast, only limited information is available about serotypes causing *K. pneumoniae* bacteremia (3,5).

Yu et al. grouped K1 and K2 serotypes and compared clinical characteristics for patients with *K. pneumoniae* bacteremia with those for patients infected with non–K1/K2 serotypes (3). Recent evidence suggests that K1 is a major cause of primary liver abscesses and has greater potential for causing metastasis, and that K2 is a major cause of secondary liver abscesses (6,8). We examined the distribution and clinical characteristics of serotypes that cause *K. pneumoniae* bacteremia from 225 patients (9) and performed PCR-based genotyping to identify capsular serotypes (10).

## The Study

The study was conducted at Far-Eastern Memorial Hospital in Taipei, Taiwan. Patients with *K*. *pneumoniae* bacteremia were identified during January 1–December 31, 2007. Identification of *K*. *pneumoniae* was based on colony morphologic features and biochemical reactions (11). Data on time until positive blood culture results were obtained from the automated blood culture system at the hospital. Data for each patient were included only once (at the time of the first detection of bacteremia). Patients <18 years of age and those not admitted to our hospital were excluded. Inactive malignancy was not included as an underlying illness. In-hospital and 14-day mortality rates were assessed. For 225 available bacterial isolates, *cps* genotyping was performed (10).

A total of 231 patients with *K*. *pneumoniae* bacteremia were observed at the hospital during the study; 225 isolates from 225 patients were used. A total of 133 (59%) of these patients had community-onset bacteremia (bacteremia identified in an emergency department). The in-hospital mortality rate was 32.4%. Among 225 isolates, 41 (18.2%) were identified as K1 serotype, 37 (16.4%) as K2, 15 (6.7%) as K57, and 8 (3.6%) as K54. The K1 serotype was found predominantly in community-onset infections (36 [87.8%] of 41 patients compared with 75 [51.0%] of 147 patients infected with non–K1/K2 serotypes; odds ratio [OR] 6.91, 95% confidence interval [CI] 2.57–18.60) (Table A1).

Underlying illness was classified as nonfatal in 75.6% of patients with K1 bacteremia (53.7% of patients with non–K1/K2 bacteremia; OR 2.67, 95% CI 1.22–5.84). A lower percentage of patients with K1 bacteremia had surgery in the previous 3 months (9.8% vs. 30.6%; OR 0.25, 95% CI 0.09–0.73). Patients with K1 bacteremia had lower mean ± SD Pittsburgh bacteremia scores than those with non–K1/K2 bacteremia (2.7 ± 3.1 vs. 4.4 ± 4.7; OR 0.90, 95% CI 0.81–0.99), but the time until a positive blood culture was obtained was not different. K1 serotype was more common in patients with liver abscesses (46.3% vs. 4.1%; OR 20.3, 95% CI 7.31–56.40) and less common in patients with urinary tract infections (UTIs) (4.9% vs. 20.4%; OR 0.20, 95% CI 0.05–0.88). The in-hospital mortality rate for patients with K1 bacteremia was lower that that for patients with non–K1/K2 bacteremia (14.6% vs. 34.7%; OR 0.32, 95% CI 0.13–0.82).

No differences were found in clinical characteristics for patients with K2 bacteremia and those with non–K1/K2 bacteremia except for a higher frequency of liver abscesses in patients with K2 bacteremia (13.5% vs. 4.1%; OR 3.67, 95% CI 1.06–12.8). For patients infected with K54 and K57 serotypes, 1 K57 serotype caused liver abscesses; no abscesses were found in patients infected with a K54 serotype. The in-hospital mortality rate was 50% (4/8) for patients with K54 bacteremia and 53.3% (8/15) for patients with K57 bacteremia.

Patients infected with a K1 serotype had lower mean ± SD Pittsburgh bacteremia scores (2.7 ± 3.1 vs. 5.0 ± 5.3; OR 0.88, 95% CI 0.78–0.98, p = 0.002) and lower 14-day and in-hospital mortality rates (9.8% vs. 27.0%; OR 0.29, 95% CI 0.08–1.03, p = 0.06; and 14.6% vs. 43.2%; OR 0.23, 95% CI 0.08–0.67, p = 0.007) than patients infected with K2 serotypes. A higher percentage of patients with K1 bacteremia had liver abscesses at the site of infection (46.3% vs. 13.5%; OR 5.53, 95% CI 1.80–17.02, p = 0.003).

Characteristics of patients with community-onset *K*. *pneumoniae* bacteremia were also analyzed (Table A2). Patients infected with a K1 serotype were more likely to have liver abscesses and less likely to have UTIs or biliary tract infections (OR 11.5, 95% CI 3.99–33.20; OR 0.20, 95% CI 0.04–0.92; and OR 0.25, 95% CI 0.07–0.91, respectively).

In our patients, K1 and K2 serotypes were found at similar frequencies (18.2% and 16.4%, respectively), which differs from results of Fung et al., in which the K1 serotype was more common (K1 30.8% and K2 5.1%) (12). Despite reported virulence of the K1 serotype, it was primarily responsible for community-onset bacteremia in patients with less severe underlying illness and associated with lower mortality rates. Moreover, the K1 serotype is associated with liver abscesses and lower mortality rates (2–7). Liver abscesses were found in 46% of patients with K1 bacteremia, and a K1 serotype was found in 63.3% of patients with liver abscesses.

## Conclusions

Management of liver abscesses has improved in Taiwan because of increased physician awareness (13). Mortality rates for patients with *K. pneumoniae* bacteremia were lower in patients with UTIs or biliary tract infections (5,14), which were less common in patients infected with a K1 serotype. Thus, patient outcomes depend more on underlying conditions and severity of sepsis than on bacterial serotypes (5,9,14).

In our previous study of the interval until a positive blood culture for *K*. *pneumoniae* bacteremia was obtained (9), we found that higher Pittsburgh bacteremia scores, a time until a positive blood culture <7 hours, and active malignancy were associated with death. In this study, we found no difference in time until a positive blood culture was obtained for patients infected with different serotypes. This interval for patients infected with K1 serotypes was slightly longer than that for patients infected with K2 and non–K1/K2 serotypes. This finding may have resulted from a higher percentage of community-onset infections and liver abscesses and less severe underlying illness in patients infected with a K1 serotype.

Studies investigating *K*. *pneumoniae* bacteremia have grouped K1 and K2 serotypes (3,7). However, such grouping may be problematic because evidence suggests that the K1 serotype is the major cause of primary liver abscesses (6). Another report showed that the genetic background of serotype K2 is diversified, and only 1 of the 2 major K2 clones was highly virulent in mice (15). These findings are consistent with our clinical observations. Differences in symptoms of patients infected with K2 and non–K1/K2 serotypes were minimal, despite slightly more liver abscesses among patients infected with K2 serotypes, which was lower than for patients infected with K1 serotypes. Because of different serotyping methods used (3,5,15), caution is required when interpreting data from various studies.

Despite greater virulence of the K1 serotype, it is predominant in patients with community-onset infections and in those with less severe underlying illness. Although the K1 serotype is the major cause of liver abscesses, it results in a lower mortality rate, which can be attributed to host factors.

## Appendix

**Table A1:** Distribution and clinical characteristics of 225 patients with *Klebsiella pneumoniae* bacteremia caused by different serotypes, Taiwan*

Characteristic	K1, n = 41	K2, n = 37	Non–K1/K2, n = 147	Univariate analysis (K1 vs. non– K1/K2)			Univariate analysis (K2 vs. non–K1/K2)	
				OR (95% CI)	p value		OR (95% CI)	p value
Age, y	61.8 ± 13.8	58.6 ± 15.1	62.2 ± 16.4	1.00 (0.98–1.02)	0.88		0.99 (0.96–1.01)	0.23
M:F	26:15	24:13	95:52	0.95 (0.46–1.95)	0.89		1.01 (0.58–2.15)	0.98
Source of patients								
Emergency department	36 (87.8)	22 (59.5)	75 (51.0)	6.91 (2.57–18.60)	<0.001		1.41 (0.68–2.93)	0.36
Ward	5 (12.2)	8 (21.6)	47 (17.0)	0.30 (0.11–0.80)	0.017		0.59 (0.25–1.38)	0.22
Intensive care unit	0	7 (18.9)	25 (32.0)	NA	NA		1.14 (0.45–2.88)	0.78
Severity of disease (McCabe classification)								
Nonfatal	31 (75.6)	24 (64.9)	79 (53.7)	2.67 (1.22–5.84)		0.014	1.59 (0.75–3.36)	0.23
Ultimately fatal	7 (17.1)	8 (21.6)	50 (34.0)	0.40 (0.17–0.97)		0.041	0.54 (0.23–1.26)	0.15
Rapidly fatal	3 (7.3)	5 (13.5)	18 (12.3)	0.57 (0.16–2.02)		0.38	1.12 (0.39–3.24)	0.84
Pittsburgh bacteremia score	2.7 ± 3.1	5.0 ± 5.3	4.4 ± 4.7	0.90 (0.81–0.99)		0.032	1.03 (0.95–1.10)	0.50
Underlying illness, condition, or value								
Diabetes mellitus	19 (46.3)	14 (37.8)	54 (36.7)	1.49 (0.74–2.99)		0.27	1.05 (0.50–2.21)	0.90
End-stage renal disease	1 (2.4)	1 (2.7)	8 (5.4)	0.43 (0.05–3.58)		0.44	0.48 (0.06–3.99)	0.50
Heart disease	4 (9.8)	8 (21.6)	33 (22.5)	0.37 (0.12–1.12)		0.08	0.95 (0.40–2.28)	0.91
Stroke	4 (9.8)	3 (8.1)	21 (14.3)	0.65 (0.21–2.01)		0.45	0.53 (0.15–1.88)	0.33
Active malignancy	5 (12.2)	6 (16.2)	29 (19.7)	0.57 (0.20–1.57)		0.27	0.79 (0.30–2.07)	0.63
Liver cirrhosis	4 (9.8)	6 (16.2)	23 (15.7)	0.58 (0.19–1.79)		0.35	1.04 (0.39–2.78)	0.93
Surgery within 3 mo	4 (9.8)	8 (21.6)	45 (30.6)	0.25 (0.08–0.73)		0.011	0.63 (0.27–1.47)	0.28
CT within 3 mo	2 (4.9)	3 (8.1)	16 (10.9)	0.42 (0.09–1.91)		0.26	0.72 (0.20–2.62)	0.62
Creatinine level, mg/dL	1.7 ± 1.9	1.6 ± 1.4	1.9 ± 2.0	0.93 (0.76–1.13)		0.45	0.91 (0.73–1.14)	0.42
Albumin level, g/dL†	2.8 ± 0.6	2.7 ± 0.6	2.7 ± 0.7	1.13 (0.57–2.23)		0.74	0.85 (0.43–1.71)	0.65
Type of infection								
Primary bacteremia	11 (26.8)	12 (32.4)	39 (26.5)	1.02 (0.47–2.22)		0.97	1.33 (0.61–2.90)	0.47
Respiratory tract	4 (9.8)	6 (16.2)	30 (20.4)	0.42 (0.14–1.28)		0.13	0.76 (0.29–1.98)	0.57
Urinary tract	2 (4.9)	3 (8.1)	30 (20.4)	0.20 (0.05–0.88)		0.033	0.34 (0.10–1.20)	0.09
Biliary tract	3 (7.3)	3 (8.1)	26 (17.7)	0.37 (0.11–1.28)		0.12	0.41 (0.12–1.44)	0.16
Liver abscess	19 (46.3)	5 (13.5)	6 (4.1)	20.3 (7.31–56.40)		<0.001	3.67 (1.06–12.8)	0.041
Intraabdominal	1 (2.4)	5 (13.5)	8 (5.4)	0.43 (0.05–3.58)		0.44	2.72 (0.83–8.85)	0.10
Catheter related	0	0	6 (4.1)	NA		NA	NA	NA
Soft tissue	0	1 (2.7)	2 (1.36)	NA		NA	2.01 (0.18–22.83)	0.57
Meningitis	1 (2.4)	2 (5.4)	0	NA		NA	NA	NA
TTP, h	14.4 ± 18.5	12.1 ± 12.1	13.1 ± 16.3	1.00 (0.99–1.02)		0.65	1.00 (0.97–1.02)	0.75
TTP<7 h	6 (14.6)	7 (18.9)	32 (21.8)	0.62 (0.24–1.59)		0.32	0.84 (0.34–2.09)	0.71
14-d mortality rate	4 (9.8)	10 (27.0)	35 (23.8)	0.35 (0.12–1.04)		0.058	1.19 (0.52–2.69)	0.68
In-hospital mortality rate	6 (14.6)	16 (43.2)	51 (34.7)	0.32 (0.13–0.82)		0.017	1.43 (0.70–2.99)	0.34
ESBL producer	0	0	4 (2.7)	NA		NA	NA (NA)	NA
Effective empirical agent	35 (85.4)	36 (97.3)	138 (93.9)	0.38 (0.13–1.14)		0.08	2.35 (0.29–19.14)	0.43

*Values are mean ± SD or no. (%) unless otherwise indicated. OR, odds ratio; CI, confidence interval; NA, not applicable; CT, computed tomography; TTP, time to positive blood culture; ESBL, extended-spectrum β-lactamase. †For K1 group, 24 patients; for K2 group, 25 patients; for non–K1/K2 group, 93 patients.

**Table A2:** Distribution and clinical characteristics of 133 patients with community-onset *Klebsiella pneumoniae* bacteremia caused by different serotypes, Taiwan*

Characteristic	K1 (n = 36)	K2 (n = 22)	Non-K1/K2 (n = 75)	Univariate analysis (K1 vs. non–K1/K2)		Univariate analysis (K2 vs. non– K1/K2)	
				OR (95% CI)	p value	OR 95% CI	p value
Age, y	60.6 ± 13.5	58.0 ± 15.6	63.7 ± 16.6	0.99 (0.96–1.01)	0.33	0.98 (0.95–1.01)	0.13
M:F	23:13	15:7	40:35	1.55 (0.68–3.51)	0.30	1.88 (0.69–5.12)	0.22
Severity of disease (McCabe classification)							
Nonfatal	29 (80.6)	18 (81.8)	51 (68.0)	1.95 (0.75–5.078)	0.17	2.12 (0.65–6.94)	0.22
Ultimately fatal	5 (13.9)	3 (13.6)	17 (22.7)	0.55 (0.19–1.63)	0.28	0.54 (0.14–2.04)	0.36
Rapidly fatal	2 (5.5)	1 (4.6)	7 (9.3)	0.57 (0.11–2.90)	0.50	0.46 (0.05–3.98)	0.48
Pittsburgh bacteremia score	2.5 ± 2.7	3.2 ± 3.7	3.8 ± 4.6	0.91 (0.81–1.03)	0.13	0.97 (0.86–1.09)	0.60
Underlying illness, condition, or value							
Diabetes mellitus	17 (47.2)	11 (50.0)	31 (41.3)	1.27 (0.57–2.83)	0.56	1.42 (0.55–3.68)	0.47
End-stage renal disease	1 (2.8)	0	5 (6.7)	0.40 (0.05–3.56)	0.41	NA	NA
Heart disease	3 (8.3)	5 (22.7)	13 (17.3)	0.43 (0.12–1.63)	0.22	1.49 (0.44–4.49)	0.57
Stroke	3 (8.3)	3 (13.6)	9 (12.0)	0.67 (0.17–2.63)	0.56	1.16 (0.29–4.71)	0.84
Active malignancy	3 (8.3)	1 (4.6)	12 (16.0)	0.48 (0.13–1.81)	0.28	0.25 (0.03–2.04)	0.20
Liver cirrhosis	3 (8.3)	3 (13.6)	9 (12.0)	0.67 (0.17–2.63)	0.56	1.16 (0.29–4.71)	0.84
Surgery within 3 mo	2 (5.6)	0	10 (13.3)	0.38 (0.08–1.85)	0.23	NA	NA
CT within 3 mo	1 (2.8)	0	3 (4.0)	0.69 (0.07–6.83)	0.75	NA	NA
Creatinine level, mg/dL	1.7 ± 2.0	1.5 ± 1.2	1.9 ± 2.1	0.95 (0.77–1.18)	0.66	0.86 (0.62–1.19)	0.35
Albumin level, g/dL†	2.7 ± 0.6	2.6 ± 0.4	2.9 ± 0.6	0.63 (0.23–1.67)	0.35	0.33 (0.09–1.27)	0.11
Type of infection							
Primary bacteremia	8 (22.2)	5 (22.7)	18 (24.0)	0.91 (0.35–2.33)	0.84	0.93 (0.30–2.88)	0.90
Respiratory tract	3 (8.3)	4 (18.2)	12 (16.0)	0.48 (0.13–1.81)	0.28	1.17 (0.34–4.06)	0.81
Urinary tract	2 (5.6)	3 (13.6)	17 (22.7)	0.20 (0.04–0.92)	0.039	0.54 (0.14–2.04)	0.36
Biliary tract	3 (8.3)	2 (9.1)	20 (26.7)	0.25 (0.07–0.91)	0.035	0.28 (0.06–1.28)	0.10
Liver abscess	18 (50.0)	5 (22.7)	6 (8.0)	11.5 (3.99–33.20)	<0.001	3.38 (0.92–12.4)	0.07
Intraabdominal	1 (2.8)	2 (9.1)	1 (1.3)	2.1 (0.13–34.80)	0.60	7.40 (0.64–85.82)	0.11
Soft tissue	0	0	1 (1.3)	NA	NA	NA	NA
Meningitis	1 (2.8)	1 (4.6)	0	NA	NA	NA	NA
TTP, h	13.6 ± 17.4	10.4 ± 3.0	15.6 ± 21.1	0.99 (0.97–1.02)	0.62	0.97 (0.90–1.04)	0.34
TTP<7 h	5 (13.9)	2 (9.1)	8 (10.7)	1.35 (0.41–4.47)	0.62	0.84 (0.34–2.09)	0.71
14-d mortality rate	3 (8.3)	3 (13.6)	13 (17.3)	0.43 (0.12–1.63)	0.22	0.75 (0.19–2.92)	0.68
In-hospital mortality rate	5 (13.9)	6 (27.3)	21 (28.0)	0.42 (0.14–1.21)	0.11	0.96 (0.33–2.80)	0.95
ESBL-producing strain	0	0	0	NA	NA	NA	NA
Effective empirical agent	31 (86.1)	22 (100.0)	72 (96.0)	0.26 (0.06–1.15)	0.08	NA	NA

*Values are mean ± SD or no. (%) unless otherwise indicated. OR, odds ratio; CI, confidence interval; NA, not applicable; CT, computed tomography; TTP, time to positive blood culture; ESBL, extended-spectrum β-lactamase. †For K1 group, 24 patients; for K2 group, 25 patients; for non–K1/K2 group, 93 patients.
